# Who Truly Benefits From First-Line Intensification in EGFR-Mutant NSCLC?

**DOI:** 10.1007/s11912-025-01723-w

**Published:** 2025-11-01

**Authors:** Michele Maffezzoli, Jason Lau, Samuel Justin, Dawood Misbah, Giuseppe Luigi Banna

**Affiliations:** 1 Department of Medicine and Surgery, University Hospital of Parma - Medical Oncology Unit, University of Parma, Parma, Italy; 2 Portsmouth Hospitals University NHS Trust, Portsmouth, PO6 3LY United Kingdom; 3 Faculty of Science and Health, School of Pharmacy and Biomedical Sciences, University of Portsmouth, Portsmouth, PO1 2EF UK; 4 University Hospital of Parma - Medical Oncology Unit, University of Parma, Parma, Italy

**Keywords:** EGFR, NSCLC, Osimertinib, Amivantamab, Lazertinib, Chemotherapy, Intensification

## Abstract

**Purpose of Review:**

The treatment landscape for advanced non-small cell lung cancer (NSCLC) with common EGFR mutations has evolved significantly, with osimertinib established as the first-line standard. Two recent phase III trials, FLAURA2 and MARIPOSA, demonstrated that first-line intensification—through osimertinib plus chemotherapy or amivantamab–lazertinib—significantly prolongs overall survival compared to osimertinib alone. The aim of this review was to critically assess whether treatment intensification should be adopted as a new standard and, if so, in which patients.

**Recent Findings:**

We identified high-risk subgroups more likely to benefit from upfront combination strategies, such as those with brain or liver metastases, L858R mutations, TP53 co-mutations, or detectable ctDNA. However, the improved efficacy comes with increased rates of grade ≥ 3 adverse events and treatment discontinuation, raising concerns about tolerability and quality of life (QoL), particularly in real-world settings with frailer patients. Post-progression strategies are also evolving and may impact the long-term value of upfront intensification. Emerging approaches, including targeted and untargeted therapies, and novel antibody–drug conjugates (ADCs), offer promising alternatives that may change future treatment options.

**Summary:**

Careful patient selection, improved toxicity management, and integration of QoL measures will be critical to ensure that the survival advantage demonstrated in trials can translate into true benefit in routine clinical practice. Future research should focus on identifying robust clinical and molecular markers to guide personalized treatment decisions.

## Introduction

The therapeutic landscape for advanced non-small cell lung cancer (NSCLC) harboring common EGFR mutations - namely exon 19 deletions (ex19del) and L858R substitutions - has significantly evolved, with osimertinib becoming the global first-line standard following the pivotal FLAURA trial. In that study, osimertinib demonstrated superior progression-free survival (PFS) and overall survival (OS) compared to first-generation EGFR TKIs (median OS: 38.6 vs. 31.8 months; HR 0.80, 95% CI: 0.65–1.00; *p* = 0.046 [1]. However, disease progression following treatment with osimertinib remains inevitable, and between 25% and 40% of patients discontinue osimertinib without receiving subsequent therapies, highlighting the need to refine first-line strategies to improve long-term outcomes [1–3]. Two recent phase III trials - FLAURA2 and MARIPOSA - have sought to improve on osimertinib monotherapy by exploring intensified upfront treatments. FLAURA2 evaluated the addition of platinum-based chemotherapy to osimertinib, while MARIPOSA investigated dual EGFR/MET inhibition with amivantamab plus lazertinib. Both trials reported statistically significant and clinically meaningful improvements in PFS and OS compared to osimertinib alone. In FLAURA2, median PFS (mPFS) was 25.5 vs. 16.7 months (HR 0.62; 95% CI: 0.49–0.79) and median OS (mOS) was 47.5 versus 37.6 months (HR 0.77; 95% CI: 0.61–0.96) [2, 4, 5]. In MARIPOSA, mPFS was 23.7 vs. 16.6 months (HR 0.70; 95% CI: 0.58–0.85), and mOS was NR vs. 36.7 months (HR 0.75, 95% CI, 0.61–0.92) [3, 6].

While these findings support the potential of first-line treatment intensification, their implementation in routine care requires careful consideration. Beyond proving superior disease control and survival over sequential strategies, any shift from osimertinib monotherapy must also ensure appropriate patient selection, manageable toxicity profiles, and a sustainable impact on healthcare resources in real-world settings [7–9].

## Efficacy in High-Risk Subgroups

Patients with baseline central nervous system (CNS) or liver metastases, L858R mutations, TP53 co-mutations, or detectable circulating tumor DNA (ctDNA) tend to have poorer outcomes, particularly when treated with osimertinib monotherapy, and may be more likely to benefit from upfront treatment intensification [10]. Exploratory subgroup analyses from both FLAURA2 and MARIPOSA suggest a consistent PFS benefit in these higher-risk populations, although such findings must be interpreted with caution due to the inherent limitations of subgroup analyses and the lack of formal interaction testing (Table 1). In contrast, among lower-risk patients, the added benefit of intensified first-line therapy appeared limited or negligible [2, 4, 6, 11–14].

**Table 1:** Comparison of selected outcomes and subgroups of patients between FLAURA2 and MARIPOSA studies

	FLAURA2			MARIPOSA		
	Osimertinib + PBC	Osimertinib	HR (95%CI)	Amivantamab + Lazertinib	Osimertinib	HR (95%CI)
Follow-up, median (months)	22.2	23.7	–	37.8	37.8	–
PFS, median (months)	25.5	16.7	0.62 (0.49–0.79)	23.7	16.6	0.70 (0.58–0.85)
OS, median (months)	47.5	37.6	0.77 (0.61–0.96)	NR	36.7	0.75 (0.61–0.92)
CNS PFS, median (months)	30.2	27.6	0.58 (0.33–1.01)	24.9	22.2	0.82 (0.62–1.09)
CNS ORR (%)	73	69	–	77	77	–
CNS CR (%)	59	43	–	–	–	–
Liver-PFS, median (months)	19.5	11.1	0.66 (0.41–1.07)	18.2	11.0	0.58 (0.37–0.91)
EGFR L858R-PFS, median (months)	21.4	13.1	0.62 (0.43–0.89)	20.3	11.1	0.65 (0.48–0.89)
TP53-PFS, median (months)	27.6	27.6	0.57 (0.29–1.12)	18.2	12.9	0.65 (0.48–0.87)
Detectable ctDNA-PFS (months)	24.8	13.9	0.60 (0.45–0.80)	20.3	14.8	0.68 (0.53–0.86)
Grade ≥ 3 AEs	64	27	-	75	43	-
Discontinuation rate (AEs), %	43	6	-	35	14	-
2nd-line therapy, %	46	60	-	74	76	-

*Abbreviations:* *PBC* Platinum-based chemotherapy, *HR* Hazard Ratio, *CI* Confidence Interval, *PFS* Progression-Free Survival, *OS* Overall survival, *CNS* Central Nervous System, *CR* Complete Response, *ORR* Objective Response Rate, *ctDNA* circulating tumor DNA, *AEs* Adverse Events, *NR* Not Reached

### CNS Metastases

The prevalence of baseline CNS metastases was comparable in FLAURA2 and MARIPOSA, affecting approximately 40% of patients [11, 15].

In FLAURA2, patients with CNS metastases experienced a notable improvement in median PFS with osimertinib plus platinum-pemetrexed versus osimertinib alone (24.9 vs. 13.8 months; HR 0.47, 95% CI 0.33–0.66) [11]. Although CNS-specific PFS data were immature, the combination arm showed a numerically longer median CNS PFS (30.2 vs. 27.6 months; HR 0.58, 95% CI 0.33–1.01), along with higher intracranial response rates, CNS overall response rate (ORR) of 73% and CR rate of 59%, compared to 69% and 43%, respectively, with monotherapy [11].

Similarly, MARIPOSA reported a median intracranial PFS of 24.9 months with amivantamab plus lazertinib versus 22.2 months with osimertinib (HR 0.82; 95% CI 0.62–1.09), with the 3-year landmark CNS PFS favoring the combination. The CNS ORR was 77% in both arms [6, 15].

These findings primarily reflect outcomes in patients with stable CNS metastases, as those with active ones are typically excluded from trials. This limits generalizability to the real-world subgroup of patients with active CNS metastases for whom evidence remains limited. It should also be acknowledged a key methodological difference between FLAURA2 and MARIPOSA regarding the brain assessment. In FLAURA2, this was performed only in patients with known brain metastases (BM) or symptoms, whereas in MARIPOSA all patients underwent baseline MRI, with repeat assessments every 24 weeks in those without BM at baseline. While the FLAURA2 strategy is closer to routine practice, systematic MRI in MARIPOSA can increase detection of asymptomatic lesions, thus influencing CNS-PFS, although the impact on OS still remains uncertain. However, an earlier intervention may reduce neurological morbidity. Whether radiotherapy for asymptomatic brain metastases should be performed upfront or deferred at progression during targeted therapy remains under investigation. Notably, the phase II TURBO-NSCLC trial demonstrated that combining EGFR TKIs with early stereotactic radiosurgery (SRS) improved time to CNS progression and local control, especially in patients with lesions ≥ 1 cm, though without an OS benefit [16] 

### Liver Metastases

Approximately 15% of patients in both FLAURA2 and MARIPOSA had liver metastases at baseline [12, 17]. In FLAURA2, median PFS was 19.5 months with osimertinib plus chemotherapy versus 11.1 months with osimertinib alone (HR 0.66; 95% CI 0.41–1.07). In MARIPOSA, the combination of amivantamab plus lazertinib yielded a median PFS of 18.2 months compared to 11.0 months with osimertinib (HR 0.58; 95% CI 0.37–0.91) [12, 17].

### L858R Mutations

Patients with L858R mutations tend to exhibit reduced sensitivity to osimertinib monotherapy [1]. In this subgroup, combination therapy significantly improved median PFS: in FLAURA2, 21.4 months with osimertinib plus chemotherapy versus 13.1 months with osimertinib alone (HR 0.62; 95% CI 0.43–0.89), and in MARIPOSA, 20.3 months with amivantamab plus lazertinib versus 11.1 months with osimertinib (HR 0.65; 95% CI 0.48–0.89) [4, 15].

### TP53 Mutations

TP53 mutations, detected in approximately 40–69% of real-world cases, are a well-recognized negative prognostic factor in EGFR-mutant advanced NSCLC, often associated with high burden of disease and presence of liver metastasis [18]. In FLAURA2, median PFS was similar in both arms at 27.6 months, although the HR favored the combination regimen (HR 0.57; 95% CI 0.29–1.12). However, it should be noted that the number of patients included in this subgroup analysis of FLAURA2 was small, limiting the robustness of this finding. In MARIPOSA, patients with TP53 co-mutations experienced improved outcomes with amivantamab plus lazertinib compared to osimertinib alone, with a median PFS of 18.2 vs. 12.9 months (HR 0.65; 95% CI 0.48–0.87) [12, 14].

### Detectable ctDNA

In FLAURA2, baseline detectable EGFR-mutant ctDNA, identified in 73% of patients, was associated with higher tumor burden and inferior prognosis. This subgroup derived greater benefit from the addition of chemotherapy, with a median PFS of 24.8 months versus 13.9 months with osimertinib monotherapy (HR 0.60; 95% CI 0.45–0.80). Conversely, patients with undetectable ctDNA had prolonged PFS irrespective of treatment, underscoring both the limited incremental value of intensification in this group and the strong prognostic significance of ctDNA status [13].

Similarly, in MARIPOSA, patients with detectable EGFR-mutant ctDNA at baseline experienced significantly longer median PFS with amivantamab plus lazertinib compared to osimertinib (20.3 vs. 14.8 months; HR 0.68; 95% CI 0.53–0.86). Among those without detectable ctDNA, about 30% of the cohort, PFS was numerically improved with the combination, though not statistically significant. Notably, the combination also conferred a PFS advantage regardless of early ctDNA clearance status at cycle 3, both in patients who cleared ctDNA (HR 0.64; 95% CI 0.48–0.87) and those who did not (HR 0.49; 95% CI 0.27–0.87) [12].

## Post-progression Strategies: Sequencing and Evolving Landscapes Beyond FLAURA2 and MARIPOSA

**Fig. 1:**
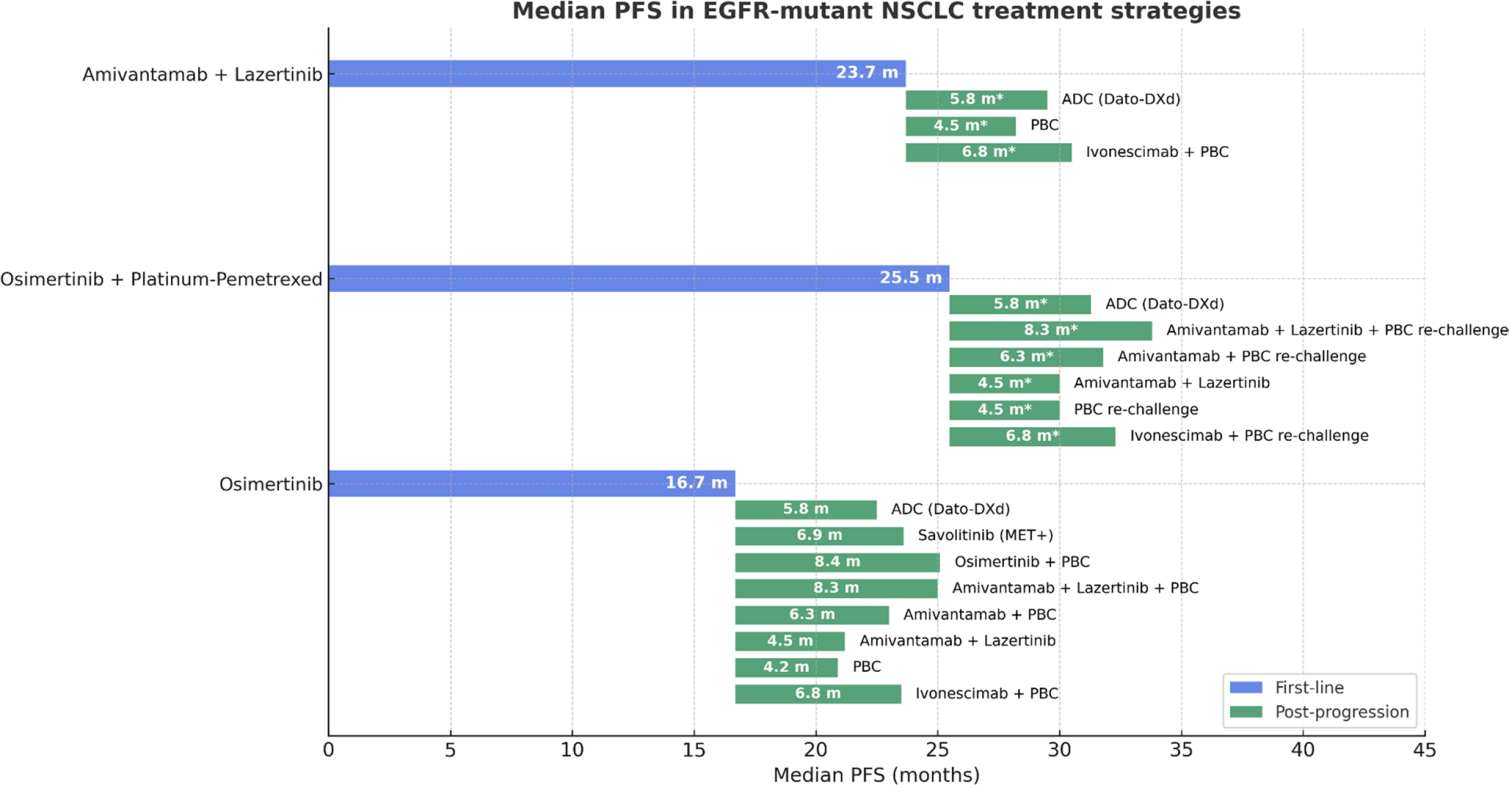
Comparison between the median PFS of different potential sequential treatment strategies for EGFR + NSCLC. Abbreviations: PBC: Platinum-based chemotherapy; PFS: Progression-Free Survival; ADC: Antibody-Drug Conjugates; Dato-Dxd: Datopotamab-deruxtecan; *: no data available, indirect comparison

Post-progression treatment pathways may differ significantly between FLAURA2 and MARIPOSA (Fig. 1).

In FLAURA2, platinum-based chemotherapy was the most frequent second-line therapy following osimertinib monotherapy. However, real-world studies suggest modest outcomes in this setting, with median PFS ranging from 3 to 6 months and OS around 10–12 months [19, 20]. Notably, 32% of patients who had already received platinum-based chemotherapy in the first-line combination arm of FLAURA2 were re-treated with platinum upon progression [2], despite limited supporting evidence for platinum re-challenge.

Looking ahead, novel combinations may enhance post-osimertinib outcomes. The CHRYSALIS-2 study demonstrated that the amivantamab–lazertinib combination yielded a median PFS of 4.5 months and a median OS of 14.8 months in patients previously treated with osimertinib [21]. Additionally, the MARIPOSA-2 trial evaluated intensification strategies after progression on EGFR TKI plus chemotherapy. Both amivantamab plus chemotherapy and amivantamab–lazertinib plus chemotherapy significantly improved PFS compared to chemotherapy alone, with median PFS of 6.3 and 8.3 months, respectively, versus 4.2 months (HR 0.48 and 0.44; p < 0.001 for both)[22]. Although these trials did not include patients who had previously received first-line osimertinib plus chemotherapy, as in FLAURA2, their findings may be relevant and merit further exploration in this population. However, intensifying second-line treatment may not be an optimal strategy, as patients progressing after first-line are often less fit and more vulnerable to toxicities.

Other post-progression strategies are also emerging after osimertinib monotherapy. One approach involves continuing osimertinib beyond progression, combined with platinum-based chemotherapy. A recent retrospective study reported a median PFS of 11 months with this strategy [20], and the randomized phase III COMPEL trial recently confirmed its potential, reaching a mPFS of 8.4 and a mOS of 15.9 months, compared with 4.4 and 9.8 months with chemotherapy alone [23].

In parallel, biomarker-guided strategies are gaining interest. Resistance profiling after osimertinib may identify actionable alterations, such as MET amplification, which can be targeted with savolitinib. This combination showed a median PFS of 6.9 months in a phase Ib trial [20, 24]. Its efficacy is currently being tested in the phase III SAFFRON study. Interestingly, FLAURA2 reported that EGFR C797S mutations and MET amplification remained the predominant resistance mechanisms regardless of treatment arm, suggesting that first-line intensification does not introduce new resistance pathways and may preserve future targeting opportunities [14].

In contrast, post-progression strategies after amivantamab–lazertinib (as in MARIPOSA) remain limited. Platinum-based chemotherapy was the most commonly administered subsequent treatment, received by 56% of patients in the experimental arm and 67% in the control arm [25]. Importantly, crossover was not permitted, and few patients received amivantamab after progression on osimertinib. Combination therapy in MARIPOSA appeared to alter resistance mechanisms: compared to osimertinib alone, the incidence of MET amplification and secondary EGFR mutations was reduced, potentially influencing subsequent treatment selection [26].

Ultimately, post-progression treatment may substantially affect overall survival and should be considered when evaluating the full clinical benefit of upfront intensification strategies in FLAURA2 and MARIPOSA.

The role of bispecific antibodies and antibody–drug conjugates (ADCs) is also emerging as a promising area. In the HARMONi trial, the combination of chemotherapy and ivonescimab, a bispecific antibody targeting PD-1 and VEGF, improved PFS compared with chemotherapy alone in patients with EGFR-mutant NSCLC progressing after 3rd-generation TKIs (6.8 vs 4.4 months; HR 0.52, 95% CI 0.41–0.66) [27]. In the TROPION-Lung05 trial, the TROP2-directed ADC datopotamab deruxtecan demonstrated an ORR of 43.6%, a median PFS of 5.8 months, and a median OS of 18.3 months in patients previously treated with both EGFR TKI and chemotherapy [28]. Similarly, the HER3-targeted ADC patritumab deruxtecan improved ORR and PFS compared to platinum-based chemotherapy in the HERTHENA-Lung02 trial, although this did not translate into an OS advantage [29]. Recently, Sacituzumab-TMT (MK-2870/SKB264), a next-generation TROP2-targeting ADC, demonstrated superior PFS compared to docetaxel in previously treated EGFR-mutant NSCLC patients, the first ADC to do so in this setting [30]. These agents may soon be evaluated in earlier lines of therapy, potentially in combination with EGFR TKIs, which could further reshape the first-line treatment paradigm.

## Toxicity and Tolerability Considerations With Combination Strategies

Both FLAURA2 and MARIPOSA demonstrated significantly higher toxicity with combination regimens, raising important concerns about tolerability, treatment burden, and quality of life (QoL). Adverse event (AE) management is particularly challenging in real-world settings, where frail and comorbid patients, often underrepresented in clinical trials, may be less able to tolerate intensified therapies. In FLAURA2, Grade ≥3 AEs occurred in 64% of patients treated with osimertinib plus chemotherapy, compared to 27% with osimertinib monotherapy. Discontinuation rates were markedly higher in the combination arm (43% vs 6%), with pemetrexed being the primary cause of treatment discontinuation (Table 1) [4, 8].

Similarly, in MARIPOSA, Grade ≥3 AEs were observed in 75% of patients receiving amivantamab–lazertinib versus 43% with osimertinib alone. Treatment discontinuation occurred in 35% of patients in the combination arm, compared to 14% in the control arm [6]. Of particular concern, venous thromboembolism (VTE) was reported in 37% of patients receiving the combination, versus only 9% with monotherapy. As a result, prophylactic anticoagulation during the first four months of therapy is recommended within clinical trials. This strategy not yet universally adopted in clinical practice, although it is increasingly considered. To improve the tolerability of the MARIPOSA regimen, enhanced supportive care strategies are essential, especially if it is to be adopted in the first-line setting. One promising development is the subcutaneous formulation of amivantamab, evaluated in the PALOMA trial, which has been shown to reduce infusion-related reactions and VTE incidence. However, skin toxicities remain common and require proactive, prophylactic management [31].

Beyond direct toxicity, combination regimens typically necessitate more frequent clinic visits compared to osimertinib monotherapy, increasing the indirect burden of care. Therefore, patient preferences and perspectives on quality of life must be integral to shared decision-making, particularly in light of the meaningful survival gains offered by these intensified treatment strategies [8].

## Conclusion

Intensified regimens in FLAURA2 and MARIPOSA have demonstrated OS benefits over osimertinib monotherapy in patients with EGFR-mutant NSCLC. As such, the future debate will not be whether to intensify, but how to personalize intensification. Indeed, these benefits come at the cost of increased toxicity, treatment burden, and healthcare resource utilization. Therefore, this strategy should currently be reserved for clinically fit patients, carefully selected based on both clinical and molecular characteristics, while placing strong emphasis on quality of life and individual patient preferences.

Whether similar survival benefits will extend to real-world populations, often older, frailer, or burdened by comorbidities and socioeconomic barriers, remains uncertain and warrants further investigation. Additionally, the emergence of effective post-progression strategies following first-line osimertinib, including targeted agents and novel drug combinations, has shown promise and may further impact OS outcomes. This evolving landscape complicates the choice of optimal treatment sequencing, which has yet to be clearly defined.

Future research should aim to identify robust molecular and clinical markers to better stratify patients and guide the decision between up-front intensification and a sequential treatment approach.

## Key References

- Yang JCH, Lu S, Hayashi H, et al. Overall Survival with Amivantamab–Lazertinib in EGFR -Mutated Advanced NSCLC. New England Journal of Medicine. Published online September 6, 2025. 10.1056/NEJMoa2503001.
  - This is the first analysis to show a survival benefit of amivantamab plus lazertinib over standard first-line osimertinib treatment.
- Planchard D, Jänne PA, Cheng Y, et al. Osimertinib with or without Chemotherapy in *EGFR* -Mutated Advanced NSCLC. New England Journal of Medicine. 2023;389(21):1935–1948. 10.1056/NEJMoa2306434.
  - This trial showed the PFS advantage of osimertinib plus chemotherapy combination over osimertinib alone. A recent press release also confirmed an OS benefit.
- La Cava G, Cortellini A, Rotow JK, Bauman JR. Navigating First-Line Treatment Options for Patients With Epidermal Growth Factor Receptor–Positive Non–Small Cell Lung Canc*er. *American Society of Clinical Oncology Educational Book. *2*025;45(3). 10.1200/EDBK-25-472784.
  - This review excellently illustrates the current treatment landscape for EGFR-mutant NSCLC.
- Felip E, Cho BC, Gutiérrez V, et al. Amivantamab plus lazertinib versus osimertinib in first-line EGFR-mutant advanced non-small-cell lung cancer with biomarkers of high-risk disease: a secondary analysis from MARIPOSA. Annals of Oncology. 2024;35(9):805–816. 10.1016/j.annonc.2024.05.541.
  - This sub-analysis of the MARIPOSA study showed improved outcomes for certain high-risk subgroups.
- Yang JC, Robichaux J, Planchard D, et al. MA12.03 FLAURA2: Resistance, and Impact of Baseline TP53 Alterations in Patients Treated With 1 L Osimertinib ± Platinum-Pemetrexed*. *Journal of Thoracic Oncology. 2024;19(10):S101-S102. 10.1016/j.jtho.2024.09.184.
  - This sub-analysis of the FLAURA2 trial reported the differential impact of the combination in patients with TP53-mutant NSCLC.

## Data Availability

No datasets were generated or analysed during the current study.
